# Supplementary material for: Exploring the return-on-investment for scaling screening and psychosocial treatment for women with common perinatal mental health problems in Malawi: Developing a cost-benefit-calculator tool
Source: PLoS One. 2024 Aug 12;19(8):e0308667. doi: 10.1371/journal.pone.0308667 (PMC11318890; doi:10.1371/journal.pone.0308667)
Supplement: S1 File — (DOCX) [file pone.0308667.s001.docx]

Supplement

Repository link for access to the data:

<https://zenodo.org/records/10533875>

DOI: 10.5281/zenodo.10533875
