# Supplementary material for: Exploring the return-on-investment for scaling screening and psychosocial treatment for women with common perinatal mental health problems in Malawi: Developing a cost-benefit-calculator tool
Source: PLoS One. 2024 Aug 12;19(8):e0308667. doi: 10.1371/journal.pone.0308667 (PMC11318890; doi:10.1371/journal.pone.0308667)
Supplement: Attachment — Submitted filename: Reviewer.docx [file pone.0308667.s002.docx]

This work develops a user-friendly decision-making tool to explore country-specific estimates for costs and economic consequences of different options for scaling screening and psychosocial interventions for women with common mental health problems in Malawi. Results suggest that positive net benefit results are highly sensitive to increased staff salaries. There are some minor concerns about the work as follows.

1. The manuscript is well-written and organized. But some sentences are difficult to follow. A native English speaker should thoroughly revise the manuscript. For instance, on page 2 – line 26, 'The aim of this study was,' maybe unnecessarily wordy. Consider replacing the noun with a corresponding verb.
2. Also, there is an absence of commas to separate a series of three or more words, phrases, or clauses, like in page 2 – line 31, page 3 – line 47, page 3 – line 63, page 3 – line 66, page 4 – line 105, page 6 – line 152, page 6 – line 153, page 6 – line 161, page 8 – line 203, page 9 – line 222, page 10 – line 273, page 11 – line 301, page 12 – line 310, page 18 – lone 397, and page 18 – line 406. Page 4 – line 81 is missing a comma before 'including.'
3. Page 4 – line 95, the noun phrase 'analysis' seems to be missing a determiner before it. Consider adding an article.
4. Page 4 – line 91, the noun phrase 'local' seems missing a determiner before it. Consider adding an article.
5. Page 4 – lines 97 and 98, consider shortening 'ways that are affordable' to 'affordable ways.'
6. Page 5 – line 113, the phrase 'who would require' may be wordy. Consider changing the wording.
7. Page 5 – line 117 seems to have an article usage problem.
8. Page 5 – line 115, the phrase 'in the form of' may be wordy. Consider changing the wording.
9. Page 5 – line 119, it appears that 'that has been' may be unnecessary in this sentence. Consider removing it.
10. Page 5 – line 120: a knowledgeable audience might find this sentence hard to read. Consider breaking it into two into 'and adapted.'
11. Page 5 – line 122, it appears that you are missing a comma before the coordinating conjunction 'and' in a compound sentence. Consider adding a comma.
12. Page 5 – lines 125 – 126, your sentence may be unclear or hard to follow. Consider rephrasing 'Next, we set out the model structure's data calculations and assumptions: what is delivered, how, by whom, the time horizon over which the delivery is modeled, and the types of costs and returns included in the model.'
13. Page 5 – line 127, the phrase 'structure of the tool' may be wordy. Consider changing the wording.
14. Page 6 – line 134, it appears that 'together with' may be unnecessary in this sentence. Consider removing it.
15. Page 7 – line 166, the noun phrase 'workforce' seems missing a determiner before it. Consider adding an article.
16. Page 7 – line 186, it appears that 'that' may be unnecessary in this sentence. Consider removing it.
17. Page 7 – line 188, it seems you're missing a comma. Consider adding a comma.
18. Page 8 – lines 195 – 197, your sentence may be unclear or hard to follow. Considering rephrasing.
19. Page 8 – line 201 seems to have an article usage problem.
20. Page 8 – line 204, page 8 – line 213, and page 9 – line 217 seems you are missing a comma. Consider adding a comma.
21. Page 8 – line 214, the noun phrase' linear increase' seems to be missing a determiner before it. Consider adding an article.
22. Page 9 – line 226, the phrase 'in a linear way' may be considered wordy. Consider changing the wording.
23. Page 9 – line 230, it seems you are missing a comma. Consider adding a comma.
24. Page 9 – line 238 appears that the phrase 'number' does not contain the correct article usage. Consider making a change.
25. Page 9 – line 243, the preposition used may be incorrect.
26. Page 9 – line 244, it seems that there is an article usage problem.
27. Page 10 – lines 145-147, your sentence may be unclear or hard to follow. Consider rephrasing.
28. Page 10 - line 255, the phrase 'Cost of training' may be wordy. Consider changing the wording.
29. Page 10 – line 252, it appears that 'number of' may be unnecessary in this sentence.
30. Page 10 – line 253, you seem to have an unnecessary comma. Consider removing the comma.
31. Page 10 – line 262, it seems you are missing a comma.
32. Page 10 – line 267, the adverbial 'also' appears misplaced in this sentence. Determine the appropriate placement for the adverb.
33. Page 10 – line 272 appears that 'those linked to' may be unnecessary in this sentence.
34. On page 11 – line 275 and page 11 – line 277, the noun phrase reduction seems to be missing a determiner before it.
35. Page 11 – line 293: a knowledgeable audience might find this sentence hard to read. Consider breaking it into two.
36. Page 12 – line 305, the verd 'reduce' does not seem to agree with the subject. Consider changing the verb form.
37. Page 14 – line 318: this sentence appears to be written in the passive voice. Consider writing in the active voice.
38. Page 15 – line 332, there seems to be a pronoun problem.
39. Page 15 – lines 335-338, this sentence appears to be written in the passive voice. Consider writing in the active voice.
40. Page 15 – line 340, the preposition used may be incorrect. Page 15 – lines 344-346, your sentence may be unclear or hard to follow. Consider rephrasing.
41. Page 15 -line 350, the preposition used may be incorrect.
42. Page 16 – line 362: a knowledgeable audience might find this sentence hard to read. Consider breaking into two.
43. Page 16 – line 367, it appears that the phrase 'total number' does not contain the correct article usage.
44. Page 16 – line 368 appears that conjunction use may be incorrect.
45. Page 16 – line 370, it appears that the phrase 'Number' does not contain the correct article usage. Consider making a change.
46. Page 18 – lines 389-391, your sentence may be unclear or hard to follow. Consider rephrasing.
47. Page 19 – line 444, the noun phrase' potential usefulness' seems to be missing a determiner before it. Consider adding an article.
48. Page 3 – line 53 shows a different use of "metal ill-heath" than, for example, on page 3, line 59, "metal -ill health." Consider replacing it with the same word in the manuscript.
49. It appears that 'decision makers' is missing a hyphen. Consider adding the hyphen(s).
50. Consider integrating a hierarchical clustering analysis into your tool to extract multiple correlation patterns from parameters and identify highly correlated data.
51. In conclusion, you never mention disadvantages and advantages compared to a machine learning strategy. In which you can predict and estimate economic consequences, but you can also obtain markers that contribute to women with common mental health problems. Consider talking about it for future research.
