# Supplementary material for: Exploring the return-on-investment for scaling screening and psychosocial treatment for women with common perinatal mental health problems in Malawi: Developing a cost-benefit-calculator tool
Source: PLoS One. 2024 Aug 12;19(8):e0308667. doi: 10.1371/journal.pone.0308667 (PMC11318890; doi:10.1371/journal.pone.0308667)
Supplement: Attachment — Submitted filename: Rebuttal letter final.docx [file pone.0308667.s003.docx]

**Rebuttal letter**

We add our response in red.

This work develops a user-friendly decision-making tool to explore country-specific estimates for costs and economic consequences of different options for scaling screening and psychosocial interventions for women with common mental health problems in Malawi. Results suggest that positive net benefit results are highly sensitive to increased staff salaries. There are some minor concerns about the work as follows.

1. The manuscript is well-written and organized. But some sentences are difficult to follow. A native English speaker should thoroughly revise the manuscript. For instance, on page 2 – line 26, 'The aim of this study was,' maybe unnecessarily wordy. Consider replacing the noun with a corresponding verb.

We changed this to: “This study sought to…”

2. Also, there is an absence of commas to separate a series of three or more words, phrases, or clauses, like in page 2 – line 31, page 3 – line 47, page 3 – line 63, page 3 – line 66, page 4 – line 105, page 6 – line 152, page 6 – line 153, page 6 – line 161, page 8 – line 203, page 9 – line 222, page 10 – line 273, page 11 – line 301, page 12 – line 310, page 18 – lone 397, and page 18 – line 406. Page 4 – line 81 is missing a comma before 'including.'

We have not changed the text in response to every one of these suggestions. Most relate to the use (or not) of the Oxford comma. Our style preference is consistent with the style used by most people who use UK English, which is *not* to use the Oxford comma.

3. Page 4 – line 95, the noun phrase 'analysis' seems to be missing a determiner before it. Consider adding an article.

We changed this sentence to: “To be useful to local and regional governments, economic analysis might need to use country- and context-specific data that reflect local infrastructure and capacities.”

4. Page 4 – line 91, the noun phrase 'local' seems missing a determiner before it. Consider adding an article.

We changed the sentence to: “Since affordability concerns together with a lack of adequate information about likely costs and benefits linked to the delivery of interventions at scale, have been major barriers to scaling PSIs in LMICs [12, 19, 20], there is a question about how economic analysis can be designed to inform strategic planning and priority-setting.

5. Page 4 – lines 97 and 98, consider shortening 'ways that are affordable' to 'affordable ways.'

Done.

6. Page 5 – line 113, the phrase 'who would require' may be wordy. Consider changing the wording.

We changed this to: “The model projects current and future populations of women *requiring* screening and PSIs, number of screenings and PSIs that are delivered, time inputs required to deliver screening and PSIs, costs and economic consequences from 2022 to 2026 at 1-year intervals.”

7. Page 5 – line 117 seems to have an article usage problem.

We changed this to: “Key parameters included as options referred to aspects of delivery for which estimates were uncertain and they had potentially high impacts on the net benefit results.”

8. Page 5 – line 115, the phrase 'in the form of' may be wordy. Consider changing the wording.

We believe it reflects the meaning of what is being said most accurately, and the choice of words is justified. No changes made.

9. Page 5 – line 119, it appears that 'that has been' may be unnecessary in this sentence. Consider removing it.

We changed this to: “We used a tool developed previously to capture the costs of implementing healthcare innovations [23] as a starting point for designing our tool.”

10. Page 5 – line 120: a knowledgeable audience might find this sentence hard to read. Consider breaking it into two into 'and adapted.'

We changed this to: “The study perspective we took was societal, meaning we included economic consequences not only as they occur to government (e.g., healthcare-related expenditure) but also to individuals (e.g., out-of-pocket expenditure). We followed approaches for valuing economic consequences (e.g., productivity losses) used in global mental health economics [22].”

11. Page 5 – line 122, it appears that you are missing a comma before the coordinating conjunction 'and' in a compound sentence. Consider adding a comma.

We separated the sentence into two sentences in line with above modifications, so this comment is no longer applicable.

12. Page 5 – lines 125 – 126, your sentence may be unclear or hard to follow. Consider rephrasing 'Next, we set out the model structure's data calculations and assumptions: what is delivered, how, by whom, the time horizon over which the delivery is modeled, and the types of costs and returns included in the model.'

We changed this to: “In the following sections, we first explain how we gathered the relevant data for the model. Then, we describe the data calculations and assumptions underlying the model. This includes details about what is delivered, how, and by whom, as well as the time horizon of the model and the types of costs and returns considered.”

13. Page 5 – line 127, the phrase 'structure of the tool' may be wordy. Consider changing the wording.

We changed this to: “Finally, we describe how the tool can be used.”

14. Page 6 – line 134, it appears that 'together with' may be unnecessary in this sentence. Consider removing it.

Done.

15. Page 7 – line 166, the noun phrase 'workforce' seems missing a determiner before it. Consider adding an article.

We changed this to: “workforce data”

16. Page 7 – line 186, it appears that 'that' may be unnecessary in this sentence. Consider removing it.

Done.

17. Page 7 – line 188, it seems you're missing a comma. Consider adding a comma.

Done.

18. Page 8 – lines 195 – 197, your sentence may be unclear or hard to follow. Considering rephrasing.

We removed the sentence as it was no longer needed after we added a reference to the Thinking Healthy Programme in the previous sentence.

19. Page 8 – line 201 seems to have an article usage problem.

We changed the sentence to: “With regards to the number and duration of sessions, we took estimates as reported in relevant publications of evaluations of PSIs and validated by local experts leading evaluations of PSIs locally [32].”

20. Page 8 – line 204, page 8 – line 213, and page 9 – line 217 seems you are missing a comma. Consider adding a comma.

Done.

21. Page 8 – line 214, the noun phrase' linear increase' seems to be missing a determiner before it. Consider adding an article.

We changed this to: “During this period, a scaling-up process is assumed starting from a zero-provision in 2022 (i.e., no screenings or PSIs are delivered and increasing linearly to what is considered the maximum possible coverage.”

22. Page 9 – line 226, the phrase 'in a linear way' may be considered wordy. Consider changing the wording.

We changed this to: “Whilst we assumed the proportion of women accessing clinics was assumed to be the same for each year, the number of women offered screening increases linearly from 80% to 100% over the course of the five years, reflecting a learning effect from health professionals, who were assumed to be able to identify more women as it becomes part of their routine.”

23. Page 9 – line 230, it seems you are missing a comma. Consider adding a comma.

The sentence has now been deleted as part of other changes to the text.

24. Page 9 – line 238 appears that the phrase 'number' does not contain the correct article usage. Consider making a change.

We changed this to: “The number of women screened positive at first stage was estimated based on the prevalence of common mental health problems (30%) and sensitivity of screening (80%). The proportion of women screened positive at second stage was estimated based on local data (Table 1).”

25. Page 9 – line 243, the preposition used may be incorrect.

We changed this to: “To estimate the costs of delivering the training, we assumed that a train-the-trainer model, also known as a cascading model, is rolled out,…”

26. Page 9 – line 244, it seems that there is an article usage problem.

See above.

27. Page 10 – lines 245-247, your sentence may be unclear or hard to follow. Consider rephrasing.

We changed this to: “In the train-the-trainer model, a lead (or ‘master’) trainer, who is a clinical psychologist, provides classroom teaching, field training and supervision. Individuals trained by the ‘master’ trainer then provide training and supervision to other trainees.”

28. Page 10 - line 255, the phrase 'Cost of training' may be wordy. Consider changing the wording.

We did not change this to ensure the distinction between cost of training and cost of supervision is clear.

29. Page 10 – line 252, it appears that 'number of' may be unnecessary in this sentence.

We changed this to: “Costs were first calculated per course and then multiplied by the number of full-time equivalents of staff who need to be employed (calculated as described above), based on a fixed number of trainees per course.”

30. Page 10 – line 253, you seem to have an unnecessary comma. Consider removing the comma.

Done. See above.

31. Page 10 – line 262, it seems you are missing a comma.

Done.

32. Page 10 – line 267, the adverbial 'also' appears misplaced in this sentence. Determine the appropriate placement for the adverb.

We changed this to: “We did not include travel costs linked to screenings since those visits would happen anyway as part of regular maternal healthcare.”

33. Page 10 – line 272 appears that 'those linked to' may be unnecessary in this sentence.

We removed this.

34. On page 11 – line 275 and page 11 – line 277, the noun phrase reduction seems to be missing a determiner before it.

We changed this to: “We calculated the healthcare savings linked to a reduction in hospital episodes for the treatment of infant diarrhoea for mothers receiving PSIs by calculating the difference in hospital episodes between intervention and control groups found in published trials and attaching the unit cost for the treatment of an episode of acute infant gastroenteritis.”

35. Page 11 – line 293: a knowledgeable audience might find this sentence hard to read. Consider breaking it into two.

We changed this, previous and following sentences to: “We calculated infants’ improved quality of life linked to a reduction in children experiencing diarrhoea because of the PSI in a similar way. We multiplied difference in diarrhoea episodes between the intervention and control group as identified by trials [34] with the average disease duration and disability weight for moderate diarrhoea. This is equivalent to the estimated DALYs averted because infants of women receiving PSIs are less likely to experience diarrhoea episodes. The DALYs-averted-per-infant estimate was multiplied by the number of women receiving PSIs.”

36. Page 12 – line 305, the verd 'reduce' does not seem to agree with the subject. Consider changing the verb form.

We changed this to: “Since children of women receiving PSIs are less likely to be stunted [34], we included this outcome in the analysis.”

37. Page 14 – line 318: this sentence appears to be written in the passive voice. Consider writing in the active voice.

Done.

38. Page 15 – line 332, there seems to be a pronoun problem.

We changed this to: “In addition, there is a worksheet ‘administration’, from which the user (technical person) can navigate to the different worksheets that present the parameters and values underlying the cost-benefit results on: population, intervention, workforce, training and supervision, travel, effectiveness, and economic consequences.”

39. Page 15 – lines 335-338, this sentence appears to be written in the passive voice. Consider writing in the active voice.

We changed this to: “Choosing from a given range of values, users (decision-makers) can change the values of the following parameters: salaries of professionals conducting the first-stage screening; salaries of professionals or volunteers conducting the second-stage screening and delivering the PSIs; setting (clinic versus community-based); group versus individual sessions.

40. Page 15 – line 340, the preposition used may be incorrect. Page 15 – lines 344-346, your sentence may be unclear or hard to follow. Consider rephrasing.

We changed the former line 340 to clarify. We also changed the former lines 344-346 to: “For the salaries, values are provided in numbers, whilst for the options concerning setting and format, options are given in proportions (0%, 25%, 50%, 75% and 100%).”

41. Page 15 -line 350, the preposition used may be incorrect.

We changed the sentence to: “The key outputs from the analysis are year-on-year estimates of (1) the costs of conducting screenings and delivering PSIs, training, supervision, and travel, and (2) economic consequences as result of treating women with PSIs including savings in healthcare expenditure, gains in productivity and reductions in DALYs.”

42. Page 16 – line 362: a knowledgeable audience might find this sentence hard to read. Consider breaking into two.

We changed this to: “For this scenario, it is assumed that first-stage screenings are provided by health professionals (e.g., midwives), who earn US$ 235 per month, and that second-stage screenings and PSIs are provided by volunteers, who receive a small payment of US$ 40 per month. It is also assumed that an equal proportion of women receive PSIs in group versus individual session format and at clinic versus community settings.”

43. Page 16 – line 367, it appears that the phrase 'total number' does not contain the correct article usage.

We added the article.

44. Page 16 – line 368 appears that conjunction use may be incorrect.

We removed ‘and’.

45. Page 16 – line 370, it appears that the phrase 'Number' does not contain the correct article usage. Consider making a change.

We removed the sentence and instead clarified in the previous sentence that ‘number’ of professionals refers to full-time equivalents.

46. Page 18 – lines 389-391, your sentence may be unclear or hard to follow. Consider rephrasing.

We changed this to: “Total estimated costs are US$ 0.66 million, which include the costs to government for employing, training and supervising staff, and paying travel reimbursements. Regarding health benefits, it is estimated that a total of 1,123 DALYs can be averted in 2022, which is equivalent to a monetary value of US$ 0.36 million.”

47. Page 19 – line 444, the noun phrase' potential usefulness' seems to be missing a determiner before it. Consider adding an article.

We added ‘the’.

48. Page 3 – line 53 shows a different use of "metal ill-heath" than, for example, on page 3, line 59, "metal -ill health." Consider replacing it with the same word in the manuscript.

We replaced mental ill-health with mental illness and also checked for the consistent use of terms.

49. It appears that 'decision makers' is missing a hyphen. Consider adding the hyphen(s).

We made sure that decision-makers and decision-making are used consistently throughout.

50. Consider integrating a hierarchical clustering analysis into your tool to extract multiple correlation patterns from parameters and identify highly correlated data.

This was beyond this exploratory analysis, but we agree that this would be useful to explore in future expansion of the analysis and tool. We added this sentence at the end of the main text: “Future analysis could make greater use of machine learning to systematically explore associations between variables and identify factors driving costs and consequences.”

51. In conclusion, you never mention disadvantages and advantages compared to a machine learning strategy. In which you can predict and estimate economic consequences, but you can also obtain markers that contribute to women with common mental health problems. Consider talking about it for future research.

We clarified in the Methods section (‘Tool description’) that simple programming with Visual Basics for Application was used.

We agree with the reviewer’s observation and – as mentioned - added the following sentence: “Future analysis could make greater use of machine learning to systematically explore associations between variables and identify factors driving costs and consequences.”
